# Few-Mode Erbium-Doped Fiber with Three-Layer Center-Recessed Doping for Gain Equalization

**DOI:** 10.3390/s25072010

**Published:** 2025-03-23

**Authors:** Shengchen Bao, Yu Cheng, Yi Tang, Ming Chen, Shijie Deng, Libo Yuan

**Affiliations:** 1 School of Optoelectronic Engineering, Guilin University of Electronic Technology, Guilin 541004, China; baosc1017@163.com (S.B.); yi_tang1999@163.com (Y.T.); mchenqq2011@163.com (M.C.); shijie.deng@guet.edu.cn (S.D.); lbyuan@vip.sina.com (L.Y.); 2 Guangxi Key Laboratory of Optoelectronic Information Processing, Guilin University of Electronic Technology, Guilin 541004, China

**Keywords:** few-mode erbium-doped fiber amplifier, gain equalization, mode division multiplexed

## Abstract

We propose a design method for few-mode erbium-doped fiber (FM-EDF) incorporating stratified doping. In the simulation design, the FM-EDF effectively reduces the differential mode gain (DMG) through the utilization of stratified doping. Simulations indicate that at an input signal power of −30 dBm, the designed fiber achieves a DMG of less than 0.5 dB across five spatial modes spanning the entire C-band (1530–1570 nm) and exceeds 20 dB gain within the range of 1530–1560 nm. Additionally, experiments using unidirectional pumping demonstrate that the FM-EDF achieves full-band gain greater than 20 dB, with a maximum gain approaching 30 dB and DMG <1 dB, across the C-band in three spatial modes. In summary, the proposed FM-EDF enhances the efficiency and reliability of long-distance signal transmission in optical communication networks, making it suitable for high-capacity optical fiber communication systems as well as long-distance sensing systems.

## 1. Introduction

Over the past three decades, with the rapid increase in the demand for high-speed and large-capacity data transmission, people have focused on optimizing optical fiber transmission systems. A variety of efficient multiplexing technologies such as time domain and frequency domain have been developed and applied, significantly improving the efficiency of information transmission. Currently, although the experimental transmission capacity of single-mode fiber has climbed to a milestone of about 100 TB/s [1,2], it faces a serious challenge: its capacity has approached the nonlinear Shannon limit, and the space for further expansion is limited [3]. In this context, space division multiplexing (SDM) technology stands out with its huge potential and becomes a key technology to break through the upper limit of communication capacity [4]. One of the carriers for SDM technology is few-mode fiber. It expands the communication channels by utilizing the parallel transmission of different linear polarization (LP) modes within the same fiber core [5]. However, long-range applications of SDM require efficient relay amplifier support. Differential mode gain (DMG) poses a significant challenge in the optimization of few-mode fiber amplifiers. It reflects the difference in gain of different modes of optical signals during amplification. Excessive DMG will aggravate the power imbalance between the channels of each mode, thus reducing the overall transmission performance [6]. This accumulation of power difference will not only increase the bit error rate at the receiving end, but also increase the risk of system interruption during long-distance transmission and multistage amplification, which seriously restricts the quality and stability of long-distance information transmission [7].

It is found that the gain equalization problem can be optimized by adjusting the refractive index distribution of the fiber, the difference in the gain medium distribution, and the spatial intensity distribution of the pump light and the signal light [8,9]. A certain degree of improvement can also be achieved by external means, such as reconfiguring the multimode pump [10,11,12]. In 2011, Jung, Y. and Alam, S. et al. put the optical signals of LP01 and LP11 modes into a 5 m two-mode fiber in the form of spatial light coupling, and achieved a gain of more than 20 dB in the band of 1550 nm~1560 nm [13]. In 2012, Kang, Q.Y. and Lim, E.L. first proposed the erbium-doped distribution of ring doping, and used it in two-mode amplification, and reduced the DMG to 2 dB through simulation [14]. Since then, these two methods have become the main means of adjusting gain balance. On this basis, many scientists have improved the doping distribution, and multi-ring doping, double-layer doping, and even three to four layers of doping have been produced [12,15,16,17]. In 2021, Zeng, Y. et al. used uniform refractive index and double-layer doping to achieve an average gain of 24.8 dB per mode at C-band with a DMG less than 0.64 dB [18]. In 2022, Liu, Y.P. et al. used double-layer doping as well as the strong coupling between modes to reduce the DMG from 9.3 to 1.1 dB in a uniformly doped step index fiber [19]. Despite significant reduction, the overall DMG remains relatively high. In 2023, Gao, J. et al. used a trench-assisted ring erbium-doped fiber amplifier with gain and a DMG greater than 20 and 1 dB [20]. But the types of spatial patterns they discussed increased to 5, and the number increased to 8.

To meet the demand for balanced relay amplification in current-mode division multiplexing (MDM) multi-channel systems and to ensure the robustness of the communication link, this research introduces a novel FM-EDF characterized by a stepped refractive index profile and a three-layer doping configuration. This design is capable of supporting the amplification of five spatial modes. Through simulation-based optimization, the optimal doping radius and concentration scheme were identified to minimize the DMG. Simultaneously, the FM-EDF is fabricated using the Modified Chemical Vapor Deposition (MCVD) method for few-mode signal amplification, achieving high gain while maintaining a low DMG. Both simulation and experimental setups employed unidirectional pumping, demonstrating advantages such as reduced DMG, a streamlined experimental configuration, and exceptional amplification performance. This design offers a viable solution to the challenge of cumulative power disparities among various channels in long-haul fiber-optic transmission systems. The findings and analyses presented in this study provide practical insights for the selection and optimization of FM-EDFA in long-distance wavelength division multiplexing transmission systems.

## 2. Simulation Principle and Design

### 2.1. Operation Principle

The theoretical model of few-mode erbium-doped fiber is based on the numerical solution of the rate and propagation equations for a two-level system of erbium ions. Mode coupling effects and transmission losses are negligible. Since the length of FM-EDF used is too short to cause obvious nonlinear damage, the influence of fiber nonlinearity in FM-EDF can be reasonably ignored [21].

Based on the energy level system, the rate equation describes the effects of absorption, stimulated radiation and spontaneous radiation on the particle population distribution of the ground state (*n*_1_) and metastable state (*n*_2_). For a two-level system with k optical signals, where the optical signal can be thought of as a specific spatial mode, the rate equation is as follows [22]:

(1) $$-\frac{dn_{1}}{dt}=\frac{dn_{2}}{dt}\sum_{k}\frac{P_{k}(z)i_{k}(r,\phi )\sigma_{a}(\nu_{k})}{h\nu_{k}}n_{1}(r,\phi ,z)-\sum_{k}\frac{P_{k}(z)i_{k}(r,\phi )\sigma_{e}(\nu_{k})}{h\nu_{k}}n_{2}(r,\phi ,z)-\frac{n_{2}(r,\phi ,z)}{\tau },$$

(2) $$n_{t}(r,\phi ,z)=n_{1}(r,\phi ,z)+n_{2}(r,\phi ,z),$$

where *h* is the Planck constant, *τ* is the metastable lifetime parameter, *ν_k_* is the frequency, and *P_k_(z)* is the power of the kth spatial mode optical signal at the fiber distance *z*. The absorption and emission cross-sections of the *k*th mode optical signal are *σ_a_* and *σ_e_*, respectively, and *n_t_* is the local erbium particle density. The normalized optical signal intensity *i_k_ (r,ϕ)* is defined as

(3) $$i_{k}(r,\phi )=\frac{I_{k}(r,\phi ,z)}{P_{k}(z)},$$

where *I_k_(r,ϕ,z)* is light intensity distribution of the *k*th beam. *P_k_(z)* is the integration of *I_k_ (r,ϕ,z)* in radial and azimuth coordinates, as follows:

(4) $$P_{k}(z)=\int_{0}^{2\pi }\int_{0}^{\infty }I_{k}(r,\phi ,z)rdrd\phi$$

The propagation equations describe the propagation of the beams through the Er-doped fiber are given by

(5) $$\begin{array}{l} \frac{dP_{k}}{dz}=u_{k}\sigma_{e}(\nu_{k})\cdot (P_{k}(z)+P_{0k})\cdot \int_{0}^{2\pi }\int_{0}^{\infty }n_{2}(r,\phi ,z)i_{k}(r,\phi )rdrd\phi \\ -u_{k}\sigma_{a}(\nu_{k})P_{k}(z)\int_{0}^{2\pi }\int_{0}^{\infty }n_{1}(r,\phi ,z)i_{k}(r,\phi )rdrd\phi , \end{array}$$

where *σ_e_* and *σ_a_* are the emission cross-section and absorption cross-section of erbium ions, respectively, which determine the absorption and emission efficiency of erbium ions towards different wavelengths of light, thereby affecting the gain of the amplifier.

Each beam propagates in the forward (u_*k*_ = 1) or backward (u_*k*_ = −1) direction, and *P_0k_* means the spontaneous emission contribution from the local metastable population *n*_2_.

(6) $$P_{0k}=mh\nu_{k}\Delta \nu_{k},$$

where the normalized number of modes is m and ∆*v_k_* is the noise bandwidth. Setting the time derivative in Equation (1) to zero and using Equation (2), the problem is reduced to the steady-state case and the ion upper-population is defined as

(7) $$n_{2}(r,\phi ,z)=n_{t}\cdot \frac{\sum_{k-1}^{n}\frac{\sigma_{a}(\nu_{k})\tau }{h\nu_{k}}i_{k}(r,\phi )P_{k}(z)}{\sum_{k-1}^{n}\frac{(\sigma_{a}(\nu_{k})+\sigma_{e}(\nu_{k}))\tau }{h\nu_{k}}i_{k}(r,\phi )P_{k}(z)+1},$$

With the specified boundary conditions at *z* = 0 and *z* = L, Equations (5) and (7) can be integrated over space, and frequency.

For FM-EDFA, the DMG value is determined by the overlap integral of the intensity distributions of the individual guided modes and the erbium doping profile. We can control the degree of overlap between the signal intensity distribution pattern of a single mode and the erbium doping profile. Therefore, the overlap integral with the signal mode can be derived [23].

(8) $$\eta_{i}=\int_{0}^{2\pi }\int_{0}^{d}\Gamma_{s,i}(r,\varphi )N_{0}(r,\varphi )\mathrm{rdrd}\varphi ,$$

In the above equation, *η* represents the overlap integral, *η_s,j_* denotes the normalized light intensity, specifically referring to the normalized intensity distribution of the guided mode relative to the cross-section of the fiber, and *N*_0_ is the doping concentration.

In the model establishment of the software, the gain and DMG of the optical signal are expressed by the following equations [24].

(9) $$G(dB)=10\log_{10}\frac{P_{out}\;(z=L)}{P_{in}(z=0)}$$

(10) $$DMG=G_{max}-G_{min}$$

### 2.2. Design of Few-Mode Erbium-Doped Fiber

**Figure 1:**
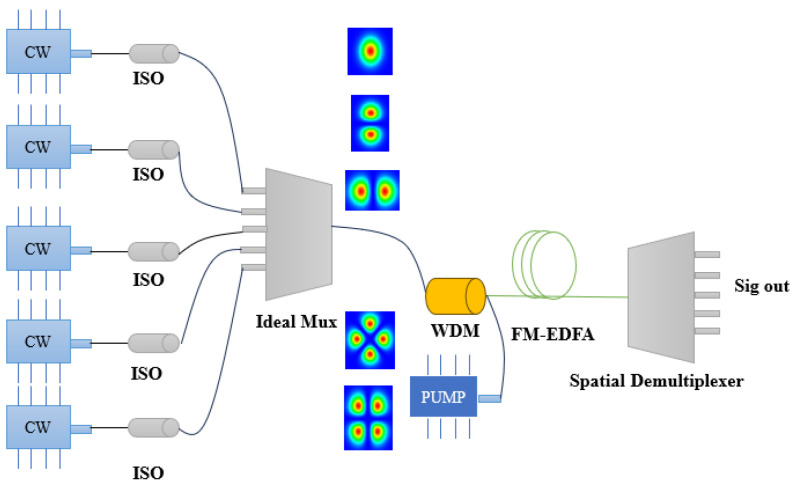
Simulated optical path (the devices in the figure from left to right are continuous wave (CW), Ideal Isolator (ISO), 980 pump laser, Ideal Mux, FM-EDFA, and Spatial Demultiplexer).

In the simulation, we used five different spatial mode signals to achieve mode multiplexing through an idealized mode multiplexing device, and amplified them in our designed few-mode erbium-doped fiber through a 980/1550 wavelength division multiplexer (WDM). After amplification, different spatial signals are demultiplexed through a spatial demultiplexing device. The structure diagram of the optical path is shown in the following Figure 1.

**Figure 2:**
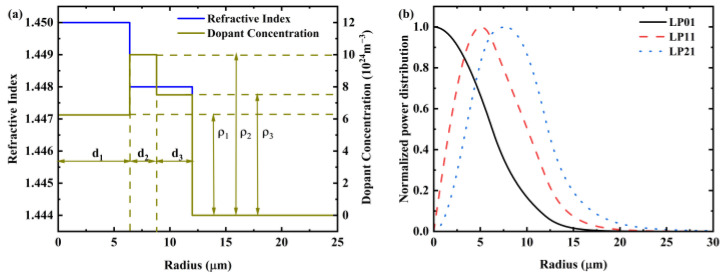
(**a**) EDP and the refractive index of the FM-EDF; (**b**) normalized power distribution with radius.

Figure 2a illustrates the refractive index and dopant distribution of the few-mode erbium-doped fiber designed by us. The fiber structure we designed comprises a core and a cladding. The fiber core consists of a high-refractive-index layer and a lower-refractive-index layer, which effectively confines multiple transmission modes within the fiber. The refractive indices for the high-refractive-index layer, the lower-refractive-index layer, and the cladding are 1.45, 1.448, and 1.444. The core radius of the FM-EDF is 12 µm, and the cladding radius is 62.5 µm, with the high-refractive-index layer extending to a radius of 6.4 µm (*d*_1_). The normalized optical intensity profiles of the modes discussed, as they propagate through the fiber, are depicted in Figure 2b.

To ensure that the overall gain of different spatial modes is consistent, it is necessary to adjust the distribution of erbium ions on the fiber profile. In general, the DMG value is determined by the overlapping integral of the strength distribution of each guide mode and the erbium-doped distribution. We can control the degree of overlap between the signal intensity distribution and the erbium-doped distribution [25]. In our designed fiber, because the signal light of different spatial modes occupies different space on the fiber interface, when these signals pass through the radial direction of the fiber, the combination of signals and erbium ions will generate corresponding mode competition, which is particularly obvious in the higher-order mode. Therefore, in general few-mode amplifier, the higher-order modes are more difficult to amplify compared to the fundamental modes. To overcome this issue, this paper adopts a three-layer erbium-doped distribution design, which reduces the erbium doping at the fiber core and increases the erbium particle concentration in the outer layers to align with the spatial distribution of higher-order modes. The erbium-doped profile (EDP) is depicted in Figure 2a.

**Figure 3:**
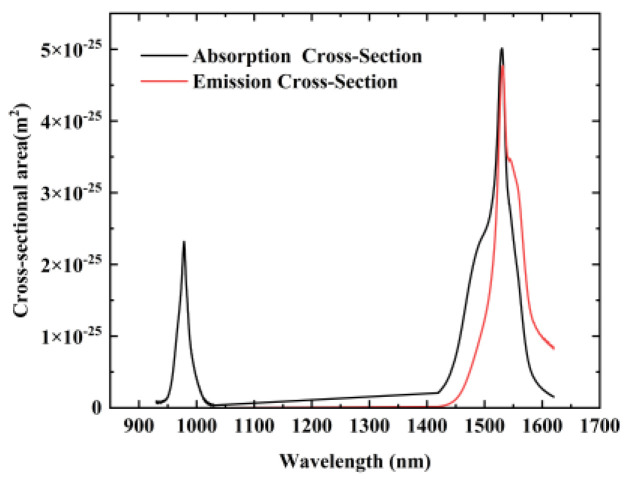
Absorption and emission spectra of erbium ions.

The erbium-doped region is divided into three layers, with *d*_1_, *d*_2_, and *d*_3_ representing the doping boundaries of the first, second, and third doping layers, respectively. Correspondingly, *ρ*_1_*, ρ*_2_, and *ρ*_3_ denote their respective doping concentrations (*d*_1_ = 6.4 µm, *d*_2_ = 2.4 µm, *d*_3_ = 3.2 µm, *ρ*_1_ = 6.25 × 10^24^ m^−3^, *ρ*_2_ = 10 × 10^24^ m^−3^, and *ρ*_3_ =7.5 × 10^24^ m^−3^). The emission cross-section (σ_e_) and absorption cross-section(σ_a_) data of erbium ions [26] are shown in Figure 3. The overlapping integration of mode intensity distribution and erbium doping distribution, as well as the metastable lifetime of erbium ions [27], are listed in Table 1.

Using Equation (8) for the calculation, we have tabulated the overlap integrals for five spatial modes as shown in Table 1. It can be observed that the overlap integral for the fundamental mode is relatively low, while the overlap integrals for the higher-order modes are closer to each other and higher than those of the fundamental mode. This is attributed to the use of core pumping, where the fundamental mode is directly amplified, whereas the higher-order modes receive less pump energy. Such a design helps to reduce DMG.

## 3. Simulation Results and Discussions

**Figure 4:**
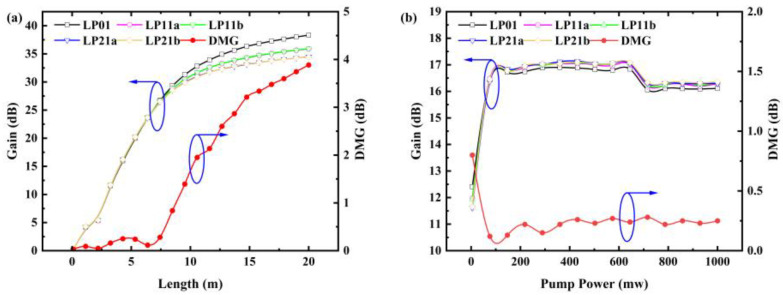
Gain and DMG vary with (**a**) fiber length and (**b**) pump light power.

When the power of the forward pumping laser at 980 nm is 450 mW and the power of input signal at 1550 nm is fixed to −30 dBm for each LP mode, the modal gain and DMG with respect to the length of the proposed FM-EDF are shown in Figure 4a.

The simulation of the gain–length relationship in the optical fibers we designed reveals an initial sharp increase in gain, followed by a gradual rise until it stabilizes as the fiber length increases. This is attributed to the increasing number of erbium ions in the fiber with length, and beyond a certain threshold length, the fiber loss and gain effects approximate, making it impossible to further amplify the optical signal. We observed that the overall trend of DMG is progressively increasing. This is due to the gradual weakening of the pump light as the distance increases, which cannot produce the same pumping effect on signal light of different modes. Additionally, as the fiber length increases, the DMG accumulates continually. When exceeding a certain critical length, the impact of the DMG becomes non-negligible, which is one of the reasons for designing Gain-Equalized FM-EDFA. In Figure 4a, we can see that DMG remains at a relatively low level within the first 7 m.

We first set the fiber length to 5 m, keeping the fiber wavelength at 1550 nm, and the input signal optical power at −30 dBm. We begin to explore the influence of pump power on the overall amplification effect. As the power of the pump signal is synchronously increased, the changes in overall gain and DMG are illustrated in Figure 4b. It is evident from Figure 4b that as the pump power increases, the gain rapidly approaches saturation. When the pump power reaches a certain high level, gain saturation is achieved at this point, and no more inverted population can be generated, and therefore, the gain of the amplifier no longer increases but decreases due to interactions between particles. Meanwhile, as the pump power increases, the DMG gradually decreases until it stabilizes, exhibiting a minimum value of 0.15 dB during this process. The initially large DMG value is due to the fact that, at the initial stage, most of the pump light is used to amplify the LP01 mode signal, with less significant amplification of other modes. As the pump power further increases, the fundamental mode signal gradually saturates, and the remaining unconsumed pump light then amplifies the signals of other modes, leading to a more stable DMG value. We intentionally reduce the erbium ion concentration at the fiber core to prevent the fundamental mode gain from becoming too prominent.

**Figure 5:**
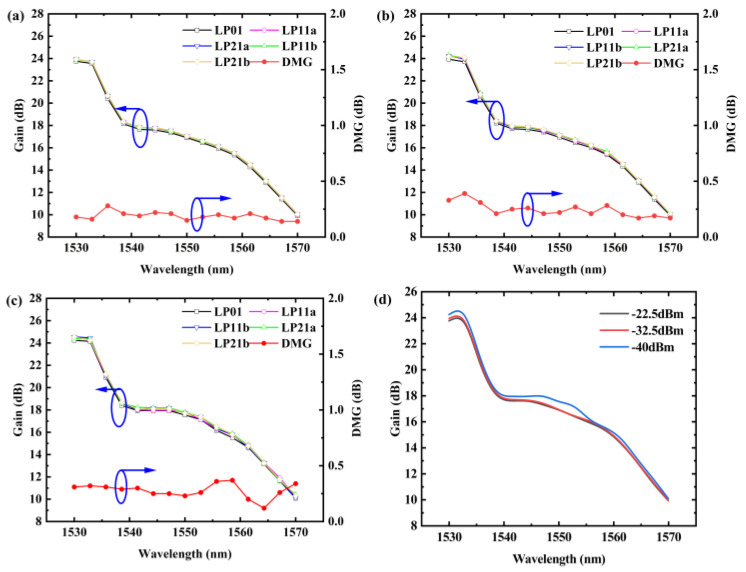
Gain and DMG at input signal optical powers of (**a**)−22.5, (**b**)−32.5, and (**c**)−40 dBm (**d**) LP01 mode gain under different input signal powers.

Subsequently, by varying the input power range of the overall signal light, we observed the gain performance of the amplifier under different application scenarios. We conducted simulations using a pump power of 450 mW and an erbium-doped fiber length of 4.5 m. In Figure 5, we simulated the gain and DMG of signal light input powers of −22.5 dBm, −32.5 dBm, and −40 dBm and listed the LP01 mode gain at different input signal powers.

We observed that the gain increased as the signal optical power decreased, while the DMG exhibited an overall trend of increasing as the signal optical energy decreased. At signal optical powers ranging from −40 to −22.5 dBm, the DMG across the entire C-band decreased to below 0.5 dB, with a minimum value of 0.1 dB. Across different small signal inputs, the DMG across the entire C-band could still be maintained below 0.5 dB, indicating the excellent applicability of our designed FM-EDFA for small signals.

**Figure 6:**
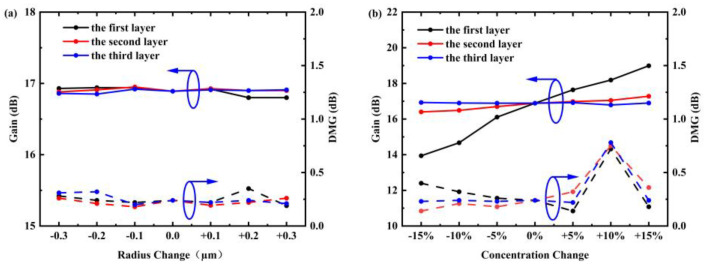
DMG and gain vary with the (**a**) doping radius and (**b**) doping concentration of each layer.

In the simulation, we also investigated the impact of manufacturing tolerances on the overall amplification performance, as illustrated in Figure 6. By analyzing the effects of variations in doping concentration and radius on the few-mode fiber (FMF), we observed that when the doping radius deviates by up to ±0.3 μm, the DMG and overall gain remain stable, with DMG consistently around 0.25 dB. However, when the doping concentration fluctuates by ±15%, significant deviations occur in both gain and DMG, with the maximum DMG increasing to 0.7 dB. Nevertheless, even under these conditions, the results still meet the requirements of current engineering applications.

## 4. Experimental Process and Result Analysis

### 4.1. Experimental Process

**Figure 7:**
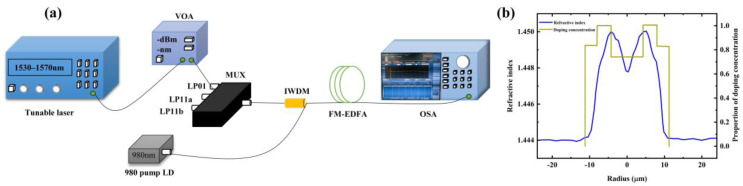
(**a**) Optical amplification measurement system of FM-EDF (**b**) The refractive index and doping distribution of few-mode optical fibers used in the experiment.

In this study, FM-EDF was fabricated via MCVD with optimized doping. In the MCVD with a solution doping technique (SDT) [28,29], porous soot without doping rare-earth ions is first deposited in the deposition tube utilizing MCVD, and then impregnated with rare-earth salt solution. After the solution was drained, the soot was dried and sintered. After drying, the porous layer was vitrified into a glass rod and sintered into a solid preform at high temperatures. Once the preform preparation was completed, it underwent a drawing process to produce optical fibers. The experimental setup in this study included a 976 nm single-mode pumped laser diode (LD) (CM96Z420-7, II-VI Photonics Pump Laser Division, Westfield Business Park, Long Road, Paignton, Devon TQ4 7AU, UK), an optical spectrum analyzer (OSA) (YOKOGAWA-AQ6370D, Yokogawa Electric Corporation, Japan), a FM-EDF, a isolator wavelength division multiplexer (IWDM), a tunable laser source (UC8714, UC Instruments, Fremont, CA 94538, USA), and a few-mode multiplexer. The experimental setup is shown in Figure 7a. Figure 7b shows the refractive index of the fiber we measured and the normalized doping distribution. The peak concentration of erbium ions is 10 × 10^24^ m^− 3^. Due to the limitations of the wavelength and power of the experimental pump laser and signal source, some situations in our simulation cannot be achieved in the experiment. Due to the limitation of laser power in the experiment, the pump power in our experiment cannot reach 1 W. The power range of the pump laser we used is 180–400 mw.

### 4.2. Experimental Results and Analysis

**Figure 8:**
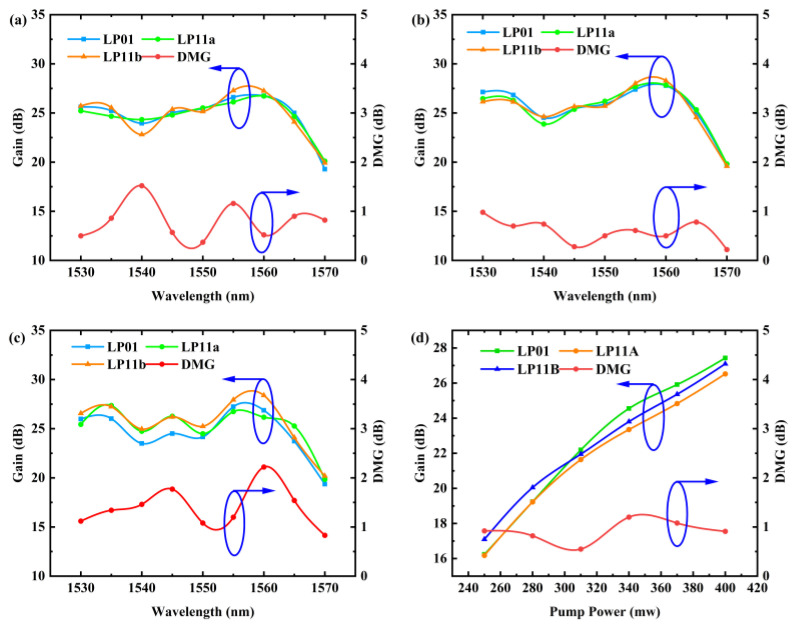
The DMG when the input signal powers are (**a**) −22.5 dBm, (**b**) −32.5 dBm, and (**c**) −40 dBm, and (**d**) when the pump power is varied.

In experimental tests, we utilized a 4.5 m length of FM-EDF to investigate the impact of various input signal powers on gain. Amplification was performed using C-band signal light with powers of −22.5 dBm, −32.5 dBm, and −40 dBm, respectively, and a 980 nm pump with a power of 400 mW. Figure 8a–c demonstrate that the 4 m FM-EDF exhibits good amplification performance. The gain slightly decreases as the input power increases. Simultaneously, the DMG followed a trend similar to our simulation results, producing larger DMG values at lower signal optical intensities. Due to the impact of fiber bending and insertion loss in the experiment, the overall DMG remained basically around 1 dB. Under different input powers, the trend of DMG changes remains generally consistent, with significant fluctuations near 1540 nm and 1565 nm. This is attributed to the emission and absorption spectra of erbium ions, which result in gain valleys and peaks at 1535 nm and 1560 nm [26], respectively, causing significant differences in gain among different modes within these two wavelength ranges.

We observed a variation in signal light gain at 1550 nm and DMG with pump power, as shown in Figure 8d. It is evident that the overall gain increases continuously with the increase in pump light intensity, which aligns with the initial simulation results. At lower pump powers, the gain of the LP01 mode remains higher among the three modes, consistent with our statement that LP01 exhibits a stronger amplification effect. As the pump power increases, the higher-order modes also begin to receive more pump excitation.

We found that the gain spectrum around 1560 nm in the simulation is not consistent with that in the experiment. Following this analysis, we believe that the main reason for the difference is that there may be some differences between the actual erbium ion doping distribution and the simulation. In addition, the experimental temperature also affects the shape of the gain spectrum, and nonlinear effects such as stimulated Raman scattering (SRS) or stimulated Brillouin scattering (SBS) are not included in the simulation. Under high-power conditions, SRS may generate additional gain in the long wavelength region (such as 1560 nm) [30]. Furthermore, the DMG also deviates from our ideal simulation results, and according to our simulated doping tolerance diagram (Figure 6b), another potential reason for this phenomenon could be an unexpected 10% increase in doping concentration compared to the original design specifications.

**Figure 9:**
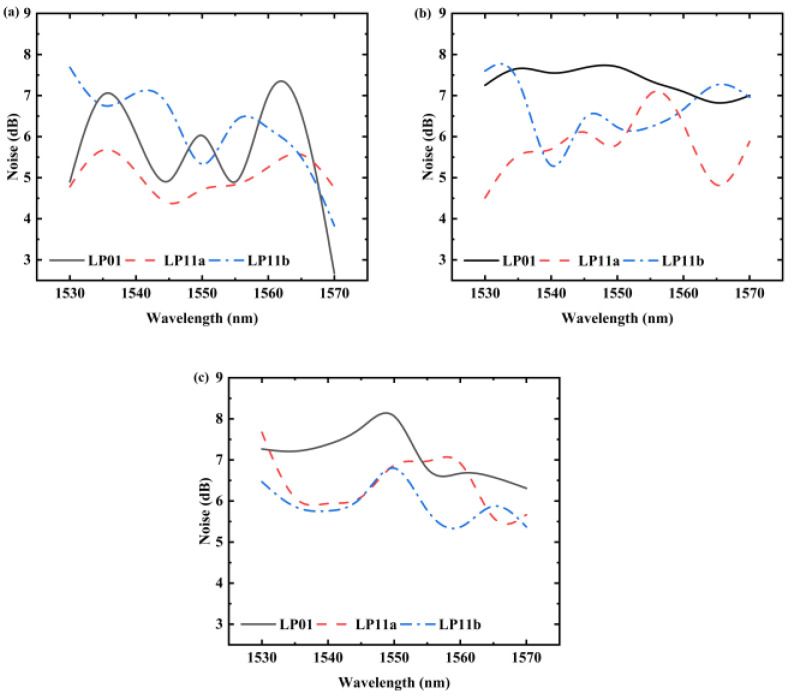
The noise at input signal powers of (**a**) −25 dBm, (**b**) −32.5 dBm, and (**c**) −40 dBm.

Figure 9 illustrates the noise characteristics observed under different input optical signal powers. Specifically, at a signal power of −25 dBm, the noise level is lower than that of other lower-power signals. The magnitude of the signal optical power influences the signal-to-noise ratio (SNR) of the amplifier noise, leading to reduced noise for higher-power input signals. In general, the noise in various input optical signal scenarios remains below 8 dB, and when the signal optical power is high, the noise can fall below 7 dB. This meets the practical engineering requirements.

In Table 2, we compare our study with the recent literature from multiple dimensions. Firstly, from the perspective of doping structure, the doping and refractive index distribution of the FM-EDFA proposed in this paper are relatively simple, simpler than the structure reported in Ref. [20]. Although Ref. [18] and [8] achieve even simpler structures, our scheme achieves a better gain and DMG performance within the same wavelength range. Secondly, in terms of noise control, our design keeps the noise below 8 dB across the entire operating wavelength range. This performance is superior to the 10 dB NF reported in Ref. [9].

## 5. Conclusions

In this study, FM-EDF was fabricated using the MCVD method with optimized doping ion concentrations. Simulations and experiments were conducted using FM-EDF under various input signal powers to optimize its performance. Simulation results indicate that with a forward pump power of 450 mW and a fiber length of 4.5 m, the FM-EDFA can achieve a maximum gain of 38 dB in the C-band with a DMG less than 0.5 dB. Experimental results demonstrate that with a forward pump power of 400 mW and a 4.5 m FM-EDF serving as the gain medium, signal amplification covering the C-band was achieved. The FM-EDFA operates effectively within the wavelength range of 1530–1570 nm. At 1555 nm, with a signal input power of −32.5 dBm, a maximum gain of 28 dB was obtained with a DMG <0.8 dB. Therefore, the proposed FM-EDF demonstrates significant potential in space-division multiplexed optical communication transmission and long-distance sensing signal amplification.

## Figures and Tables

**Table 1:** Overlapping integration of mode intensity distribution and erbium doping distribution, as well as the metastable lifetime of erbium ions.

Mode	Overlapping Integral	Er Metastable Lifetime
LP01	1.5808 × 1022	10 ms
LP11 a/b	2.2434 × 1022	
LP21 a/b	2.6548 × 1022	

**Table 2:** Comparison of FM-EDFA performance.

Ref.	Structure	Gain (dB)	DMG (dB)	NF (dB)
[18] (Simulation)	An oversized two-layer erbium ion distribution	~25	<0.64	5–7
[20] (Simulation)	Three-layer refractive index with three-layer doping	~20	~0.4	<5
[8] (Simulation)	Three-layer doping with uniform refractive index	<22	<0.5	<5
[9] (Experiment)	~	~26	≤0.6	<11
This paper (Sim)	Three-layer doping with dual-layer refractive index	<25	<0.5	~
This paper (Exp)	~	<30	<0.8	<8

## Data Availability

The raw data supporting the conclusions of this article will be made available by the authors on request.
